# Perceptions of vaping addiction and cessation among Australian young adults: A qualitative study

**DOI:** 10.18332/tid/225412

**Published:** 2026-08-10

**Authors:** Adrian Piotrowski, Reshika Chand, Becky Freeman, Christina Watts, Emily Jenkinson, Ciara Madigan, Bronwyn McGill, Shiho Rose

**Affiliations:** 1 Sydney School of Public Health, Faculty of Medicine and Health, The University of Sydney, Camperdown, New South Wales, Australia; 2 Prevention Research Collaboration, Sydney School of Public Health, Faculty of Medicine and Health, The University of Sydney, Camperdown, New South Wales, Australia; 3 Charles Perkins Centre, The University of Sydney, Camperdown, NSW, Australia; 4 The Daffodil Centre, The University of Sydney, and Cancer Council NSW, Sydney, Australia; 5 Cancer Prevention and Advocacy Division, Cancer Council NSW, Woolloomooloo, New South Wales, Australia

**Keywords:** vape or e-cigarette use, addiction, cessation, young adults, qualitative study

## Abstract

**INTRODUCTION:**

Vape use in Australian young adults (18–24 years) is the highest of all age groups. With long-term health behaviors established during young adulthood, vaping addiction is concerning, as is establishing appropriate cessation supports for this group. Our qualitative study explored the perspectives and experiences of vaping-related addiction and cessation among Australian young adults.

**METHODS:**

This study was a qualitative study using data from the Generation Vape research study. Twenty semi-structured focus groups were conducted online in February 2023. The Framework Method was used to thematically analyze for key themes and sub-themes.

**RESULTS:**

Participants (n=114) were, on average, 21 years old, approximately half were female (51.8%), and most had vaped (78.9%). Our analysis identified three themes: 1) factors facilitating regular vaping; 2) perceptions of vaping addiction; and 3) vaping cessation behaviors. Themes appeared to influence each other and highlighted the nuanced ways young adults understand addiction and reflect on their use. While young adults agreed that vapes were addictive, addiction itself was categorized as habitual (i.e. embedded in daily routine) or compulsive (i.e. an uncontrollable urge) – with varying levels of acceptability. Young adults experiencing addiction to vaping highlighted that this was an unintended outcome, describing cessation challenges of nicotine withdrawal and social temptation. Those not considering cessation felt in control of their use and would avoid addiction, viewing vaping as a transient life-phase.

**CONCLUSIONS:**

Our findings are timely considering the recent policy changes in Australia. Vaping attitudes and potential misconceptions need to be challenged in young adults who may underestimate addiction. Given that vaping is a socially embedded practice among young adults, support tailored specifically to this group is needed to effectively promote cessation.

## INTRODUCTION

Young adults (YAs, 18–24-year-olds) represent the most common age group to have tried or currently use vapes (e-cigarettes), which is disproportionate to other age groups^1^. In Australia, 21% of YAs currently vape, a fourfold increase from 2019 to 2022–2023^2^. While vapes were originally positioned as smoking cessation aids for adults, the tobacco and vaping industry promotes them to appeal to young people^3^. YA cite factors such as curiosity, flavors, and social norms as reasons for initiating vaping, rather than for smoking cessation^4^. The majority (58%) of Australian YAs who have ever vaped report having never smoked prior to vaping for the first time^2^.

Despite growing evidence that vaping increases the risk of cigarette smoking uptake^5^, YAs may underestimate the associated harms of vaping and perceive vapes as less risky than traditional cigarettes^6^. With young adulthood critical in establishing health behaviors that continue into later adulthood^7^, there is concern for YAs who develop a nicotine addiction. About one-third (32%) of YAs who currently vape report being unable to stop or reduce their vape use, despite wanting to or having tried^1^. High vaping frequency may indicate nicotine addiction^8^, with 26% of YAs who currently vape reporting daily use^1^. YAs who vape may underestimate the addictiveness of vapes^6^, particularly compared to non-users^9^.

Given the prevalence of YA vaping and concerns about addiction, encouraging and supporting cessation is important. YAs report a desire to quit vaping, with 33% having made a quit attempt and 41% intending to quit^10^, but have distinct needs which further complicate quit attempts^11^. Vape use patterns vary among YAs, with both high and low levels of vaping commonly reported^10^, Despite admitting to regular vaping, 29% of YAs do not identify as ‘vapers’^10^, suggesting they may dissociate from the behavior, decreasing quitting intentions^11^. Other vaping cessation barriers for YAs are social influences and poly-product use, including other tobacco products or marijuana alongside vaping^11^.

Australian research focused on YA vaping is limited, particularly for addiction and cessation. Cross-sectional studies report that positive social norms and ease of vape access are associated with reduced quitting intention and attempts in Australian YAs^12,13^. Qualitative research with YAs who currently or formerly vaped report that normalization of vaping, easy access and the desire to feel included impede cessation^12,13^. While addiction is also recognized as a barrier to cessation^12,13^, how YAs perceive addiction and its subsequent impact on cessation efforts remains unclear. To highlight concerns and identify strategies to prevent and reduce vaping specific to this age group, this study explored the experiences and perceptions of vaping addiction and cessation among Australian YAs.

## METHODS

### Study design

This qualitative study was part of Generation Vape^10^, an Australian research project exploring vape use and behaviors in young people and YAs. Findings from YAs are reported using the consolidated criteria for reporting qualitative research checklist^14^. Ethics approval was provided by the Human Research Ethics Committee at The University of Sydney (2021/442).

### Participant recruitment and sampling methods

Recruitment was coordinated through a professional recruitment agency using purposive sampling of YAs aged 18–24 years, from Australian metropolitan and a mix of rural and regional areas. Participants were emailed a Participant Information Statement and provided informed written consent using a link to an online Participant Consent Form. They received a $100 gift voucher (which cannot be used on tobacco or alcohol) as reimbursement for their time.

### Data collection

Data were collected in February 2023 using semi-structured online focus group discussions. Twenty focus group discussions (n=114 individual participants) were segmented by age (18–20; 21–24 years) and current vaping and smoking status: never vaped and did not currently smoke (NVNS), vaped but did not currently smoke (VNS), and vaped and currently smoked (VS) (Supplementary file Material 1). Segmentation ensured homogeneity, facilitating honest and open discussion. All groups were male or female, with non-binary participants nominating to attend their preferred groups.

Focus group discussions were conducted online using Zoom video conference software for approximately 90 minutes each. All focus group discussions were moderated by the same male facilitator with extensive experience in behavioral science and tobacco control research, and who had no prior relationship with participants. The discussion guide was developed and piloted by the Generation Vape research team and explored participants’ awareness, use, and knowledge of vaping as per project aims. This study focused on participant responses about addiction and cessation: knowledge of addiction, perceived addictiveness of vapes, attitudes towards quitting, and personal experiences or observations of friends in relation to vaping addiction and cessation (Supplementary file Material 2). Focus group discussions were video recorded with participants’ consent and their cameras on, with recordings de-identified and transcribed verbatim.

### Data analysis

Transcripts were exported to NVivo 14 and thematically analyzed^15^ using the Framework Method^16^. Two researchers (AP and RC) independently reviewed three transcripts for data familiarity and classified relevant excerpts by applying ‘codes’. Coding was performed inductively (guided by emergent patterns in the data rather than a pre-specified coding structure) due to the exploratory nature of the research. Iterative discussions between researchers (AP and RC) were used to compare codes, group them into themes and sub-themes, resolve discrepancies, and reach agreement. Where agreement could not be reached, a resolution was achieved by discussions with two other researchers (BM and SR). Once finalized, the coding framework was then applied to the remaining transcripts, which were reviewed and coded against the agreed themes and sub-themes, using quotes to support findings. The data were then charted into the framework matrix to systematically reduce the data and allow for the final stage of mapping and interpretation.

## RESULTS

### Participant characteristics

Participants were, on average, 21 years old. Approximately half (51.8%) were female, 48.2% were from the state of New South Wales, and 83.3% resided in metropolitan areas (Table 1).

**Table 1:** Characteristics of young adult focus group participants recruited across metropolitan and regional Australia, 2023 (N=114)

*Characteristics*		*n*	*%*
**Gender**	Male	53	46.5
	Female	59	51.8
	Non-binary	2	1.8
**Vaping and smoking status**	Never vaped, did not currently smoke	24	21.1
	Vaped, did not currently smoke	43	37.7
	Vaped and currently smoked	47	41.2
**Age** (years)	18–20	56	49.1
	21–24	58	50.9
**State/Territory**	Australian Capital Territory	1	0.9
	New South Wales	55	48.2
	Queensland	14	12.3
	South Australia	17	14.9
	Tasmania	10	8.8
	Victoria	17	14.9
**Place of residence**	Metropolitan	83	72.8
	Regional	31	27.2

### Focus group themes

**Figure 1:**
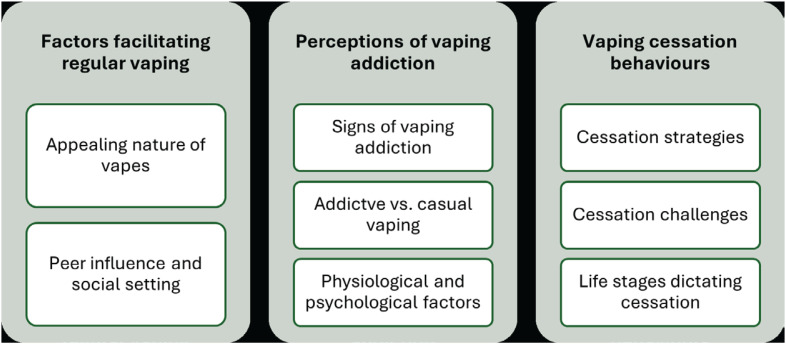
Themes and sub-themes from Australian young adult focus group participants (N=114)

Three overarching themes were identified: factors facilitating regular vaping, perceptions of vaping addiction, and vaping cessation behaviors. These themes illustrated the perceptions of and experiences with initial attraction to vaping and then to more regular and dependent use, including cessation attempts and the influencing factors of participants’ behaviors. The themes and sub-themes are illustrated in Figure 1.

### Factors facilitating regular vaping

*Appealing nature of vapes*

The appealing nature of vapes typically encouraged participants’ initial attraction. Features including the innocuous appearance and bright device colors made vaping attractive while seeming relatively harmless. Other appealing characteristics highlighted were the flavors and aromas of vapes. Collectively, these attributes facilitated regular use but also created challenges with subsequent cessation. For example, the range and variety of flavors maintained an individual’s interest in vaping and encouraged ongoing use:

*‘You just feel like a little secret agent. You go into a little sketchy shop, you do the side eye, and you’re like, “You got the iGets? You got those Gunnpods?” and they slide the list across. Then you get to pick from like a thousand different fun chemical flavors, and you get a new one every time, and you have your favorite. It’s just a fun little thing.’* (Female, 23 years, Regional, VNS)

Vapes were described as accessible, easy to use, and discreet. This convenience contributed to participants’ regular use. The social acceptability and open use in public spaces of vapes, especially compared to smoking, were also seen to encourage habitual use:

*‘Vaping is a lot more acceptable to do inside; you do it everywhere. So you’re just reaching for it without even noticing.’* (Female, 22 years, Metropolitan, VNS)

*Peer influence and social settings*

Participants described vaping as a social activity done with their friends or peers. The cohabitation of vaping while drinking alcohol was frequently discussed, particularly in social contexts, which further ingrained regular vape use. Many acknowledged the social pressure to start vaping and how this subsequently encouraged regular use. With vaping embedded in social contexts, this created challenges to refrain from vaping by those who wanted to ‘fit in’:

*‘You almost felt a bit weird, like not using it. And obviously, you want it when you quit – when people are doing it right in front of you, it’s nearly impossible not to.’* (Female, 21 years, Metropolitan, VS)

### Perceptions about vaping addiction

*Signs of vaping addiction*

Participants discussed the addictive nature of vapes and recognized the role of nicotine. Vape addiction tended to be described in two ways: 1) habitual – characterized as vape use becoming integrated into their normal routine; and 2) compulsive – described as the overwhelming need to vape and experiencing withdrawal symptoms in its absence:

*‘… when I was addicted, it was kind of like I didn’t think about it at all – it’s just kind of like blinking at that point. You don’t think about it, you just do it – when you’re on withdrawals, it’s the only thing you think about.’* (Female, 20 years, Metropolitan, NVNS)

A perceived sign of addiction included associated anxiety when unable to vape. This was recognized by non-vaping participants who observed it in their peers, with participants who vaped using words such as ‘on edge’, ‘aggravated’, or ‘angry’ to describe withdrawal symptoms. Some agreed they continued vaping to avoid the discomfort of withdrawal symptoms:

*‘I don’t find anything positive about [vapes]. I genuinely just do it to avoid the withdrawal symptoms because I find that I get super agitated if I don’t have it with me.’* (Male, 19 years, Metropolitan, VNS)

*Perceptions of addictive versus casual vaping*

Participants talked about addictive vaping as problematic, whereas casual/social vaping was not, reflecting the extremes of the perceived vaping spectrum. Those who identified as casual/social vape users perceived themselves as being in control of their vaping. Vaping addiction was seen as the individual’s responsibility and reflected an inability to responsibly manage use. The notion of ‘addictive personalities’ was raised, emphasizing that some individuals are inherently more prone to vaping addiction:

*‘I kind of see it as like a lack of self-control – it really should have been a phase when we were younger – it’s like a lifestyle now for some people, and – I’m like, “You gotta get it together now, we’re adults”.’* (Female, 20 years, Metropolitan, NVNS)

Participants who identified as casual vape users described their behavior as ‘infrequent’ or ‘inconsequential’, with little reason to quit. They believed they could easily quit when they chose to and viewed vaping as one of many risky health behaviors, de-emphasizing their vaping as low risk:

*‘Personally, I don’t do it enough where it’s causing any problems in my life – I know something’s going to kill me one day, so I’m like “nah”.’* (Female, 21 years, Metropolitan, VS)

*Physiological and psychological factors*

Vaping was seen to provide sensory gratification by participants who described enjoying ‘head spins’ and feeling ‘floaty’. However, these feelings reduced with regular vaping, and some admitted to using vapes with higher nicotine content to experience these effects:

*‘I think over time you build up a tolerance to it – I actually like the slight head spin that you get from the stronger ones.’* (Male, 24 years, Metropolitan, VNS)

Vaping was reported to help participants feel calm; they noted reliance on vapes as a strategy to alleviate stress. Some recognized an association with addiction and satisfying their nicotine cravings:

*‘There have been times when I’m maybe having a panic attack – that nicotine release running through my body makes me feel calm again. I guess the absence of the craving makes me feel calm. It’s like, I don’t know, Boomers have their tea and I have my vape.’* (Female, 23 years, Regional, VNS)

Adverse impacts on psychological wellbeing, such as feeling ‘irritable’, ‘depressed’, and ‘disappointed’ were also experienced when trying to quit vaping. Some acknowledged the role of ‘chemical changes’ resulting in these experiences and the effect on enjoyment, addiction, and cessation:

*‘Neurotransmitters in your brain, that are telling your body, “Oh my god, this is amazing” – so yes it’s a habit, but it’s a habit because of your body’s chemical reaction.’* (Female, 20 years, Metropolitan, VS)

### Vaping cessation behaviors

*Cessation strategies*

Participants who had attempted or contemplated quitting commonly attributed effects on long-term health as motivating factors. Views on using nicotine replacement therapies (NRTs) such as patches and gums for cessation support were mixed. NRT was overlooked by some as it was associated with smoking cessation and described as ‘cigarette-y’. However, positive experiences of NRT as a quit vape support were shared:

*‘I bought the patches – definitely, it cut down dramatically in the 5 days.’* (Male, 24 years, Metropolitan, VS)

Support from social networks (family and friends) and GPs was seen as important to facilitate successful quitting. Gradually reducing vaping rather than abruptly quitting, and maintaining a routine and staying busy were seen as effective approaches:

*‘Cold turkey was less successful than those who gradually reduced. I stopped buying it and then just doing it when you’re out, and then eventually stopped.’* (Female, 23 years, Metropolitan, VS)

*Cessation challenges*

Of participants who tried to quit, many described the process as difficult, with withdrawal symptoms (described in ‘signs of vaping addiction’ sub-theme) largely acknowledged as a barrier:

*‘It’s just the absence of nicotine withdrawal that I like staying away from. That’s why I continue to vape – I don’t like going through that itch phase, it’s uncomfortable.’* (Female, 23 years, Regional, VNS)

Participants perceived information and support for quitting vapes as limited compared to smoking cessation. Various reasons were offered, including low awareness of health messaging on the harms of vaping, types of cessation support, and services to assist with quitting. Individual factors such as boredom, willpower, self-doubt, and inner conflict were also attributed to failed quit attempts.

Social norms and contexts of vaping were highlighted as barriers to quitting. For example, the normalization of vaping encouraged open use around peers, making vapes difficult to escape. For some, the solution to quitting was to completely remove themselves from particular peer groups or social situations:

*‘Cutting myself off from the source, or like, from mates that do it all the time – avoiding the situation where I would get tempted.’* (Male, 24 years, Regional, VNS)

*Life stages dictating cessation*

There was a prevailing belief that participants would naturally progress to quitting, with vaping a transient life-phase. They highlighted wanting to enjoy their young years and live in the moment. Similarly, cessation was perceived as being influenced by life stages, with quitting seeming more natural and appropriate with the achievement of life milestones:

*‘When I stop going out. When I start buckling down for marriage and children, I don’t wanna be puffing a vape.’* (Male, 19 years, Metropolitan, VS)

## DISCUSSION

This qualitative study identified key experiences and perceptions of vaping addiction and cessation among Australian YAs, filling an important gap in the literature. Our findings provide early insights which highlight how supports can be tailored for YAs by considering the unique needs of this group in reducing or quitting vaping. There was agreement among YAs that vaping is addictive, however they noted varying degrees of acceptable use. Nonetheless, attitudes to vaping and addiction were nuanced, illustrating the complexities of the issue and challenges with cessation.

Vape use was described as automatic and unconscious. Increasing vaping frequency or using stronger devices were discussed as ways to satisfy cravings and feel a sense of normalcy as tolerance to nicotine grew. These findings align with domains such as ‘craving/urge to use’ and ‘automaticity’ identified in a framework for measuring vaping dependence^17^, and are consistent with symptoms of addiction and withdrawal, specifically highlighted by YAs^18^. Social acceptability and convenience of vapes, also reported by others^17^, were seen to encourage continued YA vaping while impeding the ability to track use and monitor levels of consumption.

YAs acknowledged vaping as addictive, yet appeared to have a poor understanding of addiction. Previous research found that 43% of YAs did not believe that vaping is addictive^6^ and that YAs inconsistently report perceived vaping addiction and their actual vape use^19^. We found a prevailing perception of vaping as a controllable behavior, preventable if individuals adopt ‘responsible’ vaping practices. This viewpoint was commonly expressed by (but not limited to) those who saw their vaping as social or casual. Similar to self-exempting beliefs^20^, some YAs rationalized their vaping by believing that infrequent use spared them from addiction and health risks.

Participants who recognized addiction in themselves de-emphasized and compartmentalized their vaping as ‘just one vice’, a common argument to justify use. The way addiction is portrayed to YAs can influence them to minimize their own vaping addiction. Previous JUUL-related social media posts have likened nicotine addiction to daily activities such as watching Netflix, reading books, and eating chocolate, to normalize vaping^21^. Such messages can change how YAs perceive addiction. Use of language, such as ‘habit’ rather than ‘addiction’ or ‘dependence’, was also observed in our study. Reframing nicotine addiction to more socially acceptable addictions (e.g. coffee) and reasons for use (e.g. concentration and stress relief), YAs may be attempting to distance themselves from the stigma of tobacco smoking^19^. YAs discussed the beneficial effects of nicotine as calming, relaxing, or alleviating anxiety. While causality between vaping and mental health is not established in YAs, studies have shown associations^22^, particularly compared to non-use^23^, highlighting the need to counter these perceptions.

Attitudes and experiences of cessation were diverse and appeared dependent on quit attempts. For YAs who had not tried to quit, cessation was simplified, believing that it would be easy. They viewed vaping as a transient life phase with milestones dictating when they would quit. YAs who tried or successfully quit described the social and environmental contexts that made cessation difficult^24^. The conflict between personal choice and conforming to social norms was challenging, further complicated when vaping with alcohol. Strategies like avoiding social situations to limit temptation are deemed necessary to reduce YA vaping, similarly reported by others^25^.

Among YAs who attempted to quit vaping, many perceived cessation support as limited, including not knowing which services to access, and the belief that NRT was solely for smoking cessation^11^. Studies show that awareness of and access to vaping cessation tools improve quitting success in YAs^26^. Promoting available cessation services and resources, e.g. Quitline service, for those who may benefit is needed^27,28^. Evidence-based vaping cessation interventions targeting YAs are currently limited, either under development or in the early stages of implementation^29,30^. Early studies demonstrate promise in technology-based interventions in addition to tailored support^11^.

### Implications

YAs potentially underestimate the nature, manifestation of, and vulnerability to vaping addiction. Vapes, by nature, are designed to be addictive and marketed to young people to attract new consumers^3^. This underscores the need to challenge misconceptions in YAs and promote messages that any vaping can be harmful regardless of frequency^5^. Similarly, perceptions of vaping as a strategy for stress relief are concerning and highlight the need to encourage individuals to instead seek and access evidence-based mental health supports. Public health campaigns would be a key component to raise such awareness and shift attitudes, with content that incorporates a negative emotional tone and anti-industry themes showing promise in youth and YA audiences^31^. In Australia specifically, the youth-targeted *‘Every vape is a hit to your health’* campaign has demonstrated early signs of impact in motivating quitting intentions^32^. However, mass media approaches should be considered as part of a comprehensive suite of strategies, inclusive of strong policy and enforcement efforts, to effectively encourage and support cessation^3^.

Australia’s vaping policy landscape has evolved significantly in recent years, with new legislation in 2024 limiting vape sales (regardless of nicotine content) to pharmacies only^33^. With these legislative changes, YAs are encouraged to quit vaping , with the Australian Government having committed to investing in vaping cessation support^34^. Targeted promotion and tailored messaging for YAs should be considered, with supports that would meet the needs of YAs. Our findings indicate that social and cultural settings influence both vaping behaviors and quitting intentions and practices. As such, cessation supports could better align with YA environments, such as navigating alcohol-related contexts and leveraging peer support. More broadly, policy measures can support reducing social cues for vaping among young adults, and as such, continuing to advocate for comprehensive health policy regulations (i.e. restricting supply and sales, and strong law enforcement) is important to reduce accessibility and social acceptability of vapes, and discourage use^3^.

### Strengths and limitations

This study focused on YAs, typically an underserved population in health promotion. Online focus groups are a recognized approach for qualitative data collection, allowing broad participant reach, and the large sample size in this study is a strength.

Given the inductive analysis, researcher bias is a possible limitation. We used strategies to facilitate consistency between researchers, including independent double-coding and feedback sessions to discuss discrepancies and reach consensus. Although it is a common practice, offering vouchers to focus group participants may have introduced participation bias. The generalizability of the findings may be limited given that recruitment challenges excluded some states and territories of Australia. Stratifying participants from a mix of locations enabled various geographical perspectives to be provided. Data identifying YAs from Aboriginal and Torres Strait Islander or culturally and linguistically diverse backgrounds were not collected, limiting a deeper understanding of these priority populations.

## CONCLUSIONS

YA expressed multifaceted views and experiences of vaping addiction and cessation. Vaping is a socially embedded practice which contributes to habitual and addictive use, and acts as a barrier to quitting. Self-exempting attitudes and perceptions that addiction is preventable demonstrate how YAs perceive controlling their vaping behaviors. Developing cessation supports appropriately tailored to YAs may help to denormalize vape use and address the misconceptions about addiction held by YAs. Highlighting various manifestations that can lead to addiction (e.g. cravings, framing use as unconscious or ‘responsible’) is also important to ensure anti-vaping messages resonate with YAs. Our findings are timely considering the 2024 Australian policy changes, highlighting that greater awareness of cessation services and resources is essential to effectively support YAs to quit.

## Supplementary Material



## Data Availability

The data supporting this research cannot be made available for privacy or other reasons. Data from interviews are unavailable for sharing due to ethical requirements.

## References

[1] Australian Institute of Health and Welfare. Alcohol, tobacco & other drugs in Australia; 2024. Accessed July 3, 2026. https://www.aihw.gov.au/reports/alcohol/alcohol-tobacco-other-drugs-australia/contents/summary

[2] Tobacco in Australia. 18.3 Prevalence of e-cigarette use. Cancer Council Victoria; 2025. Accessed July 3, 2026. https://www.tobaccoinaustralia.org.au/chapter-18-e-cigarettes/18-3-prevalence-of-e-cigarette-use

[3] Watts C, Rose S, McGill B, Yazidjoglou A. New image, same tactics: Global tobacco and vaping industry strategies to promote youth vaping. Health Promotion International. 2024;39(5):126. doi:10.1093/heapro/daae126 PMC11533144 39495009

[4] Jongenelis MI, Brennan E, Slevin T, et al. Factors associated with intentions to use e-cigarettes among Australian young adult non-smokers. Drug Alcohol Rev. 2019;38(5):579-587. doi:10.1111/dar.12963 31317596

[5] Banks E, Yazidjoglou A, Brown S, et al. Electronic cigarettes and health outcomes: Umbrella and systematic review of the global evidence. Med J Aust. 2023;218(6):267-275. doi:10.5694/mja2.51890 36939271 PMC10952413

[6] Jongenelis MI, Kameron C, Rudaizky D, Slevin T, Pettigrew S. Perceptions of the harm, addictiveness, and smoking cessation effectiveness of e-cigarettes among Australian young adults. Addict Behav. 2019;90:217-221. doi:10.1016/j.addbeh.2018.11.004 30447513

[7] Bonnie RJ, Stroud C, Breiner H, Committee on Improving the Health, Safety, and Well-Being of Young Adults; Board on Children, Youth, and Families; Institute of Medicine; Investing in the Health and Well-Being of Young Adults. National Academies Press (US); 2015. doi:10.17226/18869 25855847

[8] Camara-Medeiros A, Diemert L, O’Connor S, Schwartz R, Eissenberg T, Cohen JE. Perceived addiction to vaping among youth and young adult regular vapers. Tob Control. 2021;30(3):273-278. doi:10.1136/tobaccocontrol-2019-055352 32198277

[9] Cooper M, Loukas A, Harrell MB, Perry CL. College students’ perceptions of risk and addictiveness of e-cigarettes and cigarettes. J Am Coll Health. 2017;65(2):103-111. doi:10.1080/07448481.2016.1254638 27805472 PMC5278646

[10] Rose S, Chapman L, Egger S, et al. Vaping and Smoking Behaviours in Australian Young Adults: A Short Report.: Cancer Council NSW; 2024. Accessed August 8, 2026. https://www.cancercouncil.com.au/wp-content/uploads/2024/01/Vaping-and-smoking-behaviours-in-Australian-young-adults-a-short-report_30-Jan-2024.pdf

[11] Berg CJ, Krishnan N, Graham AL, Abroms LC. A synthesis of the literature to inform vaping cessation interventions for young adults. Addict Behav. 2021;119:106898. doi:10.1016/j.addbeh.2021.106898 33894483 PMC8113079

[12] Jongenelis MI, Gill M, Lawrence N, Wakefield CE. Quitting intentions and behaviours among young Australian e-cigarette users. Addiction. 2024;119(9):1608-1615. doi:10.1111/add.16530 38923180

[13] Rahman N, Sebar B, Sofija E. An exploration of the barriers and facilitators shaping vaping cessation among Australian Young Adults. Youth. 2024;4(4):1526-37. doi:10.3390/youth4040098

[14] Tong A, Sainsbury P, Craig J. Consolidated criteria for reporting qualitative research (COREQ): A 32-item checklist for interviews and focus groups. Int J Qual Health Care. 2007;19(6):349-357. doi:10.1093/intqhc/mzm042 17872937

[15] Braun V, Clarke V. Reflecting on reflexive thematic analysis. Qualitative Research in Sport, Exercise and Health. 2019;11(4):589-97. doi:10.1080/2159676X.2019.1628806

[16] Gale NK, Heath G, Cameron E, Rashid S, Redwood S. Using the framework method for the analysis of qualitative data in multi-disciplinary health research. BMC Med Res Methodol. 2013;13:117. doi:10.1186/1471-2288-13-117 24047204 PMC3848812

[17] Andreas M, Grundinger N, Wolber N, et al. Subjective experiences of the addictive potential of E-cigarettes: Results from focus group discussions. Addiction Research & Theory. 2024;32(6):432-9. doi:10.1080/16066359.2023.2288831

[18] Simpson KA, Kechter A, Schiff SJ, et al. Characterizing symptoms of e-cigarette dependence: A qualitative study of young adults. BMC Public Health. 2021;21(1):959. doi:10.1186/s12889-021-10945-z 34016066 PMC8138971

[19] Dobbs PD, Hodges EJ, Dunlap CM, Cheney MK. Addiction vs. dependence: A mixed methods analysis of young adult JUUL users. Addict Behav. 2020;107:106402. doi:10.1016/j.addbeh.2020.106402 32224428

[20] Oakes W, Chapman S, Borland R, Balmford J, Trotter L. “Bulletproof skeptics in life’s jungle”: Which self-exempting beliefs about smoking most predict lack of progression towards quitting?. Prev Med. 2004;39(4):776-782. doi:10.1016/j.ypmed.2004.03.001 15351545

[21] Czaplicki L, Kostygina G, Kim Y, et al. Characterising JUUL-related posts on Instagram. Tob Control. 2020;29(6):612-617. doi:10.1136/tobaccocontrol-2018-054824 31266903

[22] Javed S, Usmani S, Sarfraz Z, et al. A scoping review of vaping, e-cigarettes and mental health impact: depression and suicidality. J Community Hosp Intern Med Perspect. 2022;12(3):33-39. doi:10.55729/2000-9666.1053 35711397 PMC9195082

[23] Becker TD, Arnold MK, Ro V, Martin L, Rice TR. Systematic review of electronic cigarette use (vaping) and mental health comorbidity among adolescents and young adults. Nicotine Tob Res. 2021;23(3):415-425. doi:10.1093/ntr/ntaa171 32905589

[24] Amin S, Dunn AG, Laranjo L. Social influence in the uptake and use of electronic cigarettes: a systematic review. Am J Prev Med. 2020;58(1):129-141. doi:10.1016/j.amepre.2019.08.023 31761515

[25] Sanchez S, Kaufman P, Pelletier H, et al. Is vaping cessation like smoking cessation? A qualitative study exploring the responses of youth and young adults who vape e-cigarettes. Addict Behav. 2021;113:106687. doi:10.1016/j.addbeh.2020.106687 33045643

[26] Graham AL, Jacobs MA, Amato MS. Engagement and 3-month outcomes from a digital e-cigarette cessation program in a cohort of 27 000 teens and young adults. Nicotine Tob Res. 2020;22(5):859-860. doi:10.1093/ntr/ntz097 31197320 PMC7171276

[27] Tobacco in Australia. 18.11 Cessation interventions to help people quit vaping. Cancer Council Victoria; 2024; Accessed July 3, 2026. https://www.tobaccoinaustralia.org.au/chapter-18-e-cigarettes/18-11-cessation-interventions-to-help-people-quit-vaping

[28] Wu J, Benjamin EJ, Ross JC, Fetterman JL, Hong T. Health messaging strategies for vaping prevention and cessation among youth and young adults: A systematic review. Health Commun. 2025;40(3):531-549. doi:10.1080/10410236.2024.2352284 38742648 PMC11561163

[29] Palmer AM, Tomko RL, Squeglia LM, et al. A pilot feasibility study of a behavioral intervention for nicotine vaping cessation among young adults delivered via telehealth. Drug Alcohol Depend. 2022;232:109311. doi:10.1016/j.drugalcdep.2022.109311 35123362 PMC8885867

[30] Cancer Institute NSW. Pave - a vaping cessation support app. Accessed July 3, 2026. https://www.cancer.nsw.gov.au/prevention-and-screening/preventing-cancer/damaging-effects-of-vaping/pave-a-vaping-cessation-support-app

[31] Hair EC, Kreslake JM, Rath JM, Pitzer L, Bennett M, Vallone D. Early evidence of the associations between an anti-e-cigarette mass media campaign and e-cigarette knowledge and attitudes: Results from a cross-sectional study of youth and young adults. Tob Control. 2023;32(2):179-187. doi:10.1136/tobaccocontrol-2020-056047 34290134

[32] Chan L, Miller N, Rickards S, et al. ‘Every Vape is a Hit to your Health’: Evaluation of an anti-vaping campaign in New South Wales, Australia. Tob Control. doi:10.1136/tc-2025-059887 42135203

[33] Australian Government Department of Health and Aged Care. New laws for vapes; 2024. Accessed July 3, 2026. https://www.health.gov.au/vaping/new-laws

[34] Department of Health and Aged Care. New supports to quit vaping and smoking Australian Government; 2024. Accessed July 3, 2026. https://www.health.gov.au/ministers/the-hon-mark-butler-mp/media/new-supports-to-quit-vaping-and-smoking

