# Supplementary material for: Perceptions of vaping addiction and cessation among Australian young adults: A qualitative study
Source: Tob Induc Dis. 2026 Aug 10;24:10.18332/tid/225412. doi: 10.18332/tid/225412 (PMC13455872; doi:10.18332/tid/225412)
Supplement: Supplementary file 1 [file TID-24-143-s1.pdf]

Tobacco Induced Diseases

Supplementary file

© 2026 Piotrowski A. et al.

DOI:
10.18332/tid/225412

The content has been provided by the author(s) and has not been reviewed, verified, or endorsed by European Publishing. It may not have undergone peer review. The views, opinions, and recommendations expressed are solely those of the author(s) and do not necessarily reflect the position of European Publishing. European Publishing accepts no responsibility or liability for any consequences arising from the use of, or reliance on, this content.

Supplementary file Material 1. Focus group characteristics and segmentation

Focus group #	Sample segment			Total (n)
	Age group	Gender	Vaping/smoking status	
1	18-20	All	Never	6
2	21-24	All	Never	6
3	18-20	Male	Vape, Never smoked	4
4	18-20	Female	Vape, Never smoked	6
5	21-24	Male	Vape, Never smoked	6
6	21-24	Female	Vape, Never smoked	5
7	18-20	Male	Vape and smoke	6
8	18-20	Female	Vape and smoke	6
9	21-24	Male	Vape and smoke	6
10	21-24	Female	Vape and smoke	5
11	18-20	All	Never	6
12	21-24	All	Never	6
13	18-20	Male	Vape and smoke	6
14	18-20	Female	Vape and smoke	6
15	21-24	Male	Vape and smoke	6
16	21-24	Female	Vape and smoke	6
17	18-20	Male	Vape, Never smoke	4
18	18-20	Female	Vape, Never smoke	6
19	21-24	Male	Vape, Never smoke	5
20	21-24	Female	Vape, Never smoke	7

Supplementary file Material 2. Generation Vape Discussion Guide: 18-24 years

Vaping –awareness/attitudes
- When did you first hear/see a vape?
- What did you first think about them?
- Have you ever tried vaping?
- What is your main reason for not vaping? What else?

Vaping – use and behaviour
- Do your friends vape?
- How acceptable is vaping amongst your friends?
- What has changed about where and who your friends vape with? Why

Health awareness and perception - vaping
- What are your thoughts about the health effects of vaping?

Nicotine and addiction
- Nicotine – what do you know about the effect of nicotine on young people?
- What do you know about addiction?
- Do you think vaping is addictive in young people? What makes you say that?
- Do you know young people who might be addicted?
- If you do, what are they wanting to do about it?

Vaping – cessation
- Do you want to quit vaping? Why/why not?
- Have you ever tried to quit vaping?
- Do you think you could quit vaping?
- Do you think vapes can help people to quit smoking?

Tobacco –awareness/attitudes
- What do you think about tobacco smoking?
- Have you ever tried cigarettes?

Vaping or Tobacco
- How do you think e-cigarettes/vapes are different to cigarettes?
- Is it the same/different being a smoker and a vaper? Why/why not?
  Prompt: health differences, social acceptability, where they can be used, etc.

Messaging & Campaigns
- Have you seen anything about vaping in the media?
- Do you think young adults are well informed about vaping or smoking?

Consolidated criteria for reporting qualitative studies (COREQ): 32-item checklist

Item	Guide questions/description	Reported on Page #
**Domain 1: Research team and reflexivity**		
Personal Characteristics		
1. Inter viewer/facilitator	Which author/s conducted the interview or focus group?	N/A
2. Credentials	What were the researcher's credentials? E.g. PhD, MD	N/A
3. Occupation	What was their occupation at the time of the study?	N/A
4. Gender	Was the researcher male or female?	Page 2
5. Experience and training	What experience or training did the researcher have?	Page 2
Relationship with participants		
6. Relationship established	Was a relationship established prior to study commencement?	Page 2
7. Participant knowledge of the interviewer	What did the participants know about the researcher? e.g. personal goals, reasons for doing the research	Page 2
8. Interviewer characteristics	What characteristics were reported about the interviewer/facilitator? e.g. Bias, assumptions, reasons and interests in the research topic	N/A
**Domain 2: study design**		
Theoretical framework		
9. Methodological orientation and Theory	What methodological orientation was stated to underpin the study? e.g. grounded theory, discourse analysis, ethnography, phenomenology, content analysis	Page 3
Participant selection		
10. Sampling	How were participants selected? e.g. purposive, convenience, consecutive, snowball	Page 2
11. Method of approach	How were participants approached? e.g. face-to-face, telephone, mail, email	Page 2
12. Sample size	How many participants were in the study?	Page 2
13. Non-participation	How many people refused to participate or dropped out? Reasons?	N/A
Setting		
14. Setting of data collection	Where was the data collected? e.g. home, clinic, workplace	Page 2
15. Presence of non-participants	Was anyone else present besides the participants and researchers?	Page 2
16. Description of sample	What are the important characteristics of the sample? e.g. demographic data, date	Page 3
Data collection		
17. Interview guide	Were questions, prompts, guides provided by the authors? Was it pilot tested?	Page 2-3
18. Repeat interviews	Were repeat interviews carried out? If yes,	N/A

	how many?	
19. Audio/visual recording	Did the research use audio or visual recording to collect the data?	Page 3
20. Field notes	Were field notes made during and/or after the interview or focus group?	N/A
21. Duration	What was the duration of the interviews or focus group?	Page 2
22. Data saturation	Was data saturation discussed?	N/A
23. Transcripts returned	Were transcripts returned to participants for comment and/or correction?	N/A
**Domain 3: analysis and findings**		
Data analysis		
24. Number of data coders	How many data coders coded the data?	Page 3
25. Description of the coding tree	Did authors provide a description of the coding tree?	N/A
26. Derivation of themes	Were themes identified in advance or derived from the data?	Page 3
27. Software	What software, if applicable, was used to manage the data?	Page 3
28. Participant checking	Did participants provide feedback on the findings?	N/A
Reporting		
29. Quotations presented	Were participant quotations presented to illustrate the themes/findings? Was each quotation identified? e.g. participant number	Page 3-6
30. Data and findings consistent	Was there consistency between the data presented and the findings?	Page 6-7
31. Clarity of major themes	Were major themes clearly presented in the findings?	Page 4
32. Clarity of minor themes	Is there a description of diverse cases or discussion of minor themes?	Page 3-6
